# Melanoma Cells Treated with GGTI and IFN-γ Allow Murine Vaccination and Enhance Cytotoxic Response against Human Melanoma Cells

**DOI:** 10.1371/journal.pone.0009043

**Published:** 2010-02-03

**Authors:** Guillaume Sarrabayrouse, Christine Pich, Raphaël Moriez, Virginie Armand-Labit, Philippe Rochaix, Gilles Favre, Anne-Françoise Tilkin-Mariamé

**Affiliations:** 1 Département «Innovation thérapeutique et Oncologie Moléculaire», INSERM U563 CPTP, Toulouse, France; 2 Institut Claudius Regaud, Toulouse, France; 3 Université Paul Sabatier, Toulouse, France; 4 Neuro-Gastroenterology and Nutrition Unit, INRA, Toulouse, France; Centre de Recherche Public de la Santé (CRP-Santé), Luxembourg

## Abstract

**Background:**

Suboptimal activation of T lymphocytes by melanoma cells is often due to the defective expression of class I major histocompatibility antigens (MHC-I) and costimulatory molecules. We have previously shown that geranylgeranyl transferase inhibition (done with GGTI-298) stimulates anti-melanoma immune response through MHC-I and costimulatory molecule expression in the B16F10 murine model [1].

**Methodology/Principal Findings:**

In this study, it is shown that vaccination with mIFN-gand GGTI-298 pretreated B16F10 cells induces a protection against untreated tumor growth and pulmonary metastases implantation. Furthermore, using a human melanoma model (LB1319-MEL), we demonstrated that *in vitro* treatment with hIFN-γ and GGTI-298 led to the up regulation of MHC-I and a costimulatory molecule CD86 and down regulation of an inhibitory molecule PD-1L. Co-culture experiments with peripheral blood mononuclear cells (PBMC) revealed that modifications induced by hIFN-γ and GGTI-298 on the selected melanoma cells, enables the stimulation of lymphocytes from HLA compatible healthy donors. Indeed, as compared with untreated melanoma cells, pretreatment with hIFN-γ and GGTI-298 together rendered the melanoma cells more efficient at inducing the: i) activation of CD8 T lymphocytes (CD8+/CD69+); ii) proliferation of tumor-specific CD8 T cells (MelanA-MART1/TCR+); iii) secretion of hIFN-γ; and iv) anti-melanoma specific cytotoxic cells.

**Conclusions/Significance:**

These data indicate that pharmacological treatment of melanoma cell lines with IFN-γ and GGTI-298 stimulates their immunogenicity and could be a novel approach to produce tumor cells suitable for vaccination and for stimulation of anti-melanoma effector cells.

## Introduction

Anti-tumor immunotherapies can be roughly subdivided into two types: passive or active. Passive immunotherapies involve the use of anti-tumor antibodies [2] or adoptive strategies using the re-infusion of a patient's lymphocytes after an *in vitro* stimulation [3]. Active immunotherapies include the injection of tumor-associated antigen (TAA) peptides [4] [5], antigen-pulsed dendritic cells (DC) [6], tumor-DC hybrids [7] or irradiated allogeneic tumor cells [8]. To be really efficient these active immunotherapies should induce not only effector cells able to immediately kill tumor cells but also memory effector cells able to prolong the protective response. The efficiency of irradiated autologous tumor cells in active immunotherapy is often limited due to their weak immunogenicity, and in particular, the frequent down-regulation of membrane class I MHC (MHC-I) antigens and/or TAA expression [9]. One of several strategies used to increase tumor immunogenicity and enhance anti-tumor T-cell response is the use of tumor cells genetically modified to over-express MHC-I and/or costimulatory molecules [8] [10]. However, these strategies are difficult to adapt to large-scale human clinical trials.

Instead, pharmacological treatments capable of increasing tumor immunogenicity have been sought [11] [12]. GGTI-298 is a pharmacological inhibitor of geranylgeranyl transferase type I enzyme. This enzyme covalently binds geranylgeranyl pyrophosphate, an isoprenoid compound of the mevalonate pathway, to numerous proteins of the Ras super family including Rho proteins. This posttranslational binding is required for the activity of a large scale of proteins [13], and geranylgeranyl transferase type I inhibition by GGTI-298 is known to inhibit growth [14], cell cycle [15] or migration in several tumor models [16]. Furthermore, it was recently shown that a novel GGTI inhibitor (P61A6) significantly suppress *in vivo* tumor growth, in a human pancreatic cancer xenograft model in mice [17]. We previously demonstrated [1] that murine B16F10 melanoma cells present a modified phenotype following *in vitro* treatment by mIFN-γ in association with GGTI-298, characterized by an up regulation of MHC-I and the GGTI-induced expression of CD80 and CD86 costimulatory molecules. Moreover, this treatment enables the T-cell-induced reduction of tumor growth in syngeneic immunocompetent mice and enhanced levels of B16F10 specific CD8 T lymphocytes TRP-2/H-2K^b^ TCR^+^ [1].

These results may provide the basis for new therapeutic strategies that would involve the *ex vivo* pharmacological treatment of melanoma cells with IFN-γ and GGTI-298 to produce useful cells for vaccination or adoptive transfer therapies. However, two essential points remained to be tested. First, in the murine model whether these pharmacological treatments produce immunogenic melanoma cells useful for *in vivo* vaccination. Then, to test in a suitable model whether these treatments are also efficient with human melanoma cells. In human models the evaluation of the increased immunogenicity of the treated melanoma cells have to be done *in vitro*. Therefore, it must be tested by co-cultures with HLA compatible lymphocytes whether these treatments would sufficiently modify the human melanoma cells to enable the induction of specific anti-tumor cytotoxic effector cells.

In the present study, we therefore used a human melanoma cell line (LB1319-MEL) expressing the EAAGIGILTV peptide, obtained from MelanA/MART-1 proteins, on HLA-A0201 molecules. These cells are particularly suitable for pre-clinical trials, since: i) HLA-A0201 is the most frequent MHC-I haplotype expressed in the Caucasian population; ii) MelanA/MART-1 protein is expressed on a large proportion of human melanoma cells [18]; and iii) the EAAGIGILTV peptide represents a prime target for immunotherapeutic protocols [19].

Our results show in the B16F10 murine model, that pharmacologically treated melanoma cells become useful tools for *in vivo* vaccination, inducing protection against tumor growth and pulmonary metastasis implantation. Furthermore, *in vitro* treatment of LB1319-MEL cells with hIFN-γ and GGTI-298 induces modifications in these cells which enable the stimulation *in vitro* of co-cultivated HLA-A0201 compatible peripheral blood mononuclear cells (PBMC) from healthy donors (HD). This stimulation induces the enhancement of tumor-specific CD8 T cells, the production of IFN-γ and the enhancement of the cytotoxic response. These results suggest that our treatments could be a novel strategy to enhance melanoma immunogenicity and to produce cells possibly useful for immunotherapies.

## Materials and Methods

### Tumor Cell Lines and Animals

LB1319-MEL human melanoma cell line was kindly provided by Professor T. Boon (Ludwig Institute for Cancer Research, Brussels). Human leukemic Jurkat cells and B16F10 murine melanoma cell line were purchased from the American Type Culture Collection. These tumor cell lines were maintained in culture by serial passages in culture medium composed of RPMI 1640 medium (Gibco-BRL) supplemented with 10% FCS, 1 mM glutamine, 100 U/mL penicillin, and 100 mg/mL streptomycin sulfate (Invitrogen Life Technologies). Cell lines were periodically tested to be mycoplasm-free. Six- to 8-wk-old female C57Bl/6 mice were obtained from Elevages Janvier. Mice were maintained in pathogen-free conditions, and studies were performed in accordance with local ethical guidelines.

### *In Vitro* Pharmacological Treatment of Tumor Cells

Melanoma cells (1×10^5^) were cultivated for 4 days in 6-well plates (Dutsher) in 2 mL of culture medium. Murine or human IFN-γ (mIFN-γ or hIFN-γ) (50 UI/mL) (Roche) and GGTI-298 (10 µM) (Calbiochem) were added alone or in combination.

### Vaccination and Analysis of Murine Tumor Growth

Naïve C57BL/6 mice were vaccinated by a single sub-cutaneous (s.c.) injection with 2×10^5^ irradiated (100 Gy) B16F10 cells. Before irradiation these melanoma cells were *in vitro* pretreated during 4 days with mIFN-γ (50 IU/mL), GGTI-298 (10 µM) or both products and then washed twice to completely remove the unfixed products. One month after this vaccination, mice were challenged and checked for s.c. tumor growth and for pulmonary metastases implantation. To study the tumor growth, mice received a tumor challenge with 1×10^5^ live untreated B16F10 cells injected sub-cutaneously. Animals were monitored for tumor growth every 3 days by palpation, and diameters of the tumors were measured using a Vernier caliper. Tumor-bearing animals were sacrificed when the tumors displayed severe ulceration or reached a size of 400 mm^2^. In the case of tumor-free mice, experiments were terminated 9 weeks after tumor challenge. All experiments included 6–8 mice/group. To study pulmonary metastases implantation, vaccinated mice were injected intravenously with 5×10^4^ live untreated B16F10 cells and sacrificed 15 days later. Macroscopic metastases were detected visually and microscopic metastases were quantified. The lungs were fixed in formalin (24 h), then slidded vertically and paraffin imbedded. Four µm sections were routinely stained by hemalun and eosin. The number of metastases was assessed and reported to the lung surfaces. The experiments on mice have been done in the appropriate conditions of husbandry, experimentation and care, because our animal house is controlled by an ethic comity. And our protocols were validated by the Ethic comity of the Institut Claudius Regaud under the control of the Regional Comity of Midi-Pyrénées (France). They received the agreement number : ICR-2009-0019.

### Flow Cytometric Analyses

For the flow cytometric analyses, PE-conjugated anti-HLA-ABC, anti-HLA-A0201, anti-HLA-DPDQDR, anti-TAP1, anti-TAP2, anti-CD69, anti-CD86, anti-PD-1L and anti-CD107a monoclonal antibodies were purchased from BD Pharmingen. FITC-conjugated anti-CD8 and PE-conjugated MART-1-HLA-A2 and EBV-HLA-A2 tetramers were purchased from Beckman Coulter. Stained cells were analyzed on a FACS Calibur (BD Pharmingen). To evaluate membrane antigen expression, the index of specific fluorescence (ISF) was determined. This was calculated using the following formula: (Median Fluorescence Intensity (MFI) with the specific antibody – MFI with the isotype control/MFI with the isotype control).

### Western Blot Analysis

Protein extracts were prepared by the standard procedure and then separated (40 µg protein/lane) on SDS-PAGE gels. Proteins were blotted onto PVDF membranes. The filters were incubated at 4°C overnight with primary antibodies against TAP1, TAP2, Tapasin, Pa28β, LMP10 (Santa Cruz Biotech) and LMP2 or LMP7 (Abcam). Actin was used as a loading control. The membranes (Bio-Rad) were then incubated with horse radish peroxidase-labeled secondary antibody (Jackson Immuno Research) for 1 h at room temperature and then detected by chemiluminescence.

### *In Vitro* Stimulation of PBMC from Healthy Donors

Volunteer healthy donors (HD) were leukapheresed after informed consent. The purification of PBMC was done by Histopaque (Sigma) density gradient centrifugation. PBMC from HLA-0201+ donors were selected and then twice stimulated *in vitro* for 7 days in 24-well plates (Dutsher) at 37°C in a 5% CO_2_ humidified atmosphere in culture medium. These stimulations were performed by incubating 2×10^6^ PBMC with 2×10^4^ irradiated (100 Gy) LB1319-MEL melanoma cells pretreated with hIFN-γ (50 IU/mL) and/or GGTI-298 (10 µM). Before co-culture, the pretreated tumor cells were washed twice to completely remove the hIFN-γ and GGTI-298. During the second stimulation and after 3 days of co-culture, 50 IU/mL of human IL-2 (hIL-2r, Proleukin, Chiron) was added.

### Analysis of Cytokine Secretion during *In Vitro* Stimulation of PBMC

Concentrations of cytokines (IL-2, IFN-γ, IL-4 and IL-10) were assayed in culture supernatant using the Bio-Plex suspension array system (Bio-Rad). Cytokine levels were measured using Bio-Plex human cytokine assays according to the manufacturer's instructions. Data were analyzed using the Bio-Plex Manager software version 2.0. Analyses were performed in triplicate and the results expressed in pg of cytokines/mL of supernatant.

### Analysis of Cytotoxic Activity by Cytofluorometry

3×10^6^ LB1319-MEL or Jurkat target cells were stained green (FL-1) with PKH67 (Sigma-Aldrich) and then washed twice to completely remove the fluorescent reagent. Stained target cells were then diluted at 5×10^3^ cells per 50 µL of culture medium. The effector cells (E) were incubated with the stained target cells (T) for 4 h at 37°C at different E/T ratios: 0,2∶1; 0,6∶1; 6∶1; 20∶1 and 60∶1 in 96–well plates (Dutsher) in triplicate. The supernatant of each well was then discarded and 150 µL of culture medium containing propidium iodine (PI, FL-3) at 50 µg/mL was added. Cytotoxicity was analyzed immediately on a FACS Calibur (BD Pharmingen). The percentage of cytotoxicity was calculated as the total number of dead target cells, stained both green (FL-1+) and red (FL-3+), divided by the total number of target cells (all green cells). The following equation was used: 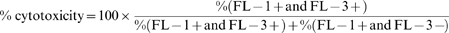



## Results

### Vaccination of Syngeneic Mice with mIFN-γ and GGTI-298 Pretreated B16F10 Cells Induces Resistance to Untreated Tumor Growth

**Figure 1:**
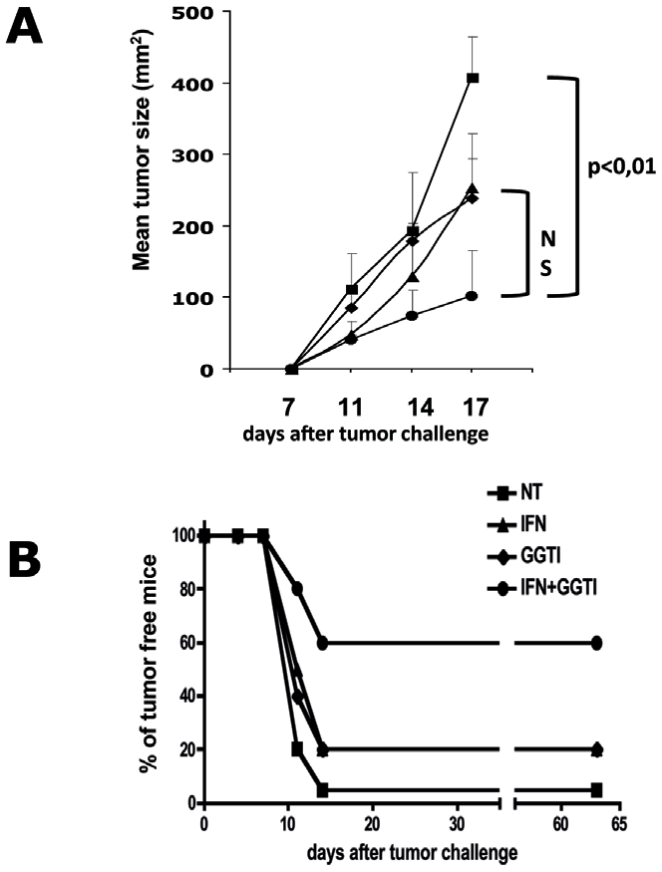
Vaccination of syngeneic mice with mIFN-γ+GGTI-298 pretreated B16F10 cells induces resistance to untreated tumor growth. C57BL/6 mice were vaccinated with untreated B16F10 cells ; mIFN-γ (50 IU/mL); GGTI-298 (10 µM); or mIFN-γ (50 IU/mL) and GGTI-298 (10 µM) pretreated B16F10 cells. (**A**) One month after vaccination tumor growth was measured after s.c. injection of 1×10^5^ live B16F10 cells, by Vernier caliper every 3 days. Groups of 6 or 7 C57Bl/6 mice were tested. Data are representative of three independent experiments. (**B**) The number of tumor free mice were calculated for each kind of vaccination. Results are pooled for the three independent experiments.

To test whether pharmacological treatment of melanoma cells with IFN-γ and GGTI-298 could produce suitable cells for vaccination a murine model was used: B16F10 cell line, a poorly immunogenic and MHC-I negative melanoma of spontaneous origin, was injected in the syngeneic C57Bl/6 mice. These melanoma cells were chosen because we have previously shown that pretreatment of B16F10 cells with mIFN-γ associated with GGTI-298 stimulates their immunogenicity through MHC-I and costimulatory molecules expression and thereby their ability to generate specific CD8 T lymphocytes [1]. To evaluate whether this pharmacologically pretreated B16F10 cells were able to elicit resistance to challenge with untreated tumor cells, mice were vaccinated once s. c. with 2×10^5^ irradiated (100 Gy) and pretreated cells and challenged s.c. with 1×10^5^ B16F10 cells one month later. The efficiency of vaccination by B16F10 cells pretreated with medium alone (NT), mIFN-γ (50 UI/mL) (IFN), GGTI-298 (10 µM) (GGTI) or mIFN-γ (50 UI/mL) associated with GGTI-298 (10 µM) (IFN+GGTI) were compared. The mice of these 4 groups were monitored every 3 days after tumor challenge, and the tumor surfaces were measured. The results showed that vaccination with pretreated B16F10 cells, induce protection against tumor growth in all groups but that the vaccination with mIFN-γ plus GGTI-298 was much more efficient as 60% of mice in this group remained tumor free two months after challenge (Figure 1B). Furthermore, tumors grow significantly slower in this group as compared to tumors in the other groups (Figure 1A). These results show that *in vitro* pretreatment of B16F10 melanoma cells with mIFN-γ and GGTI-298 render them immunogenic cells usable for vaccination protocols.

### Vaccination with mIFN-γ and GGTI-298 Pretreated B16F10 Cells Induces Resistance to Pulmonary Metastases Implantation

**Figure 2:**
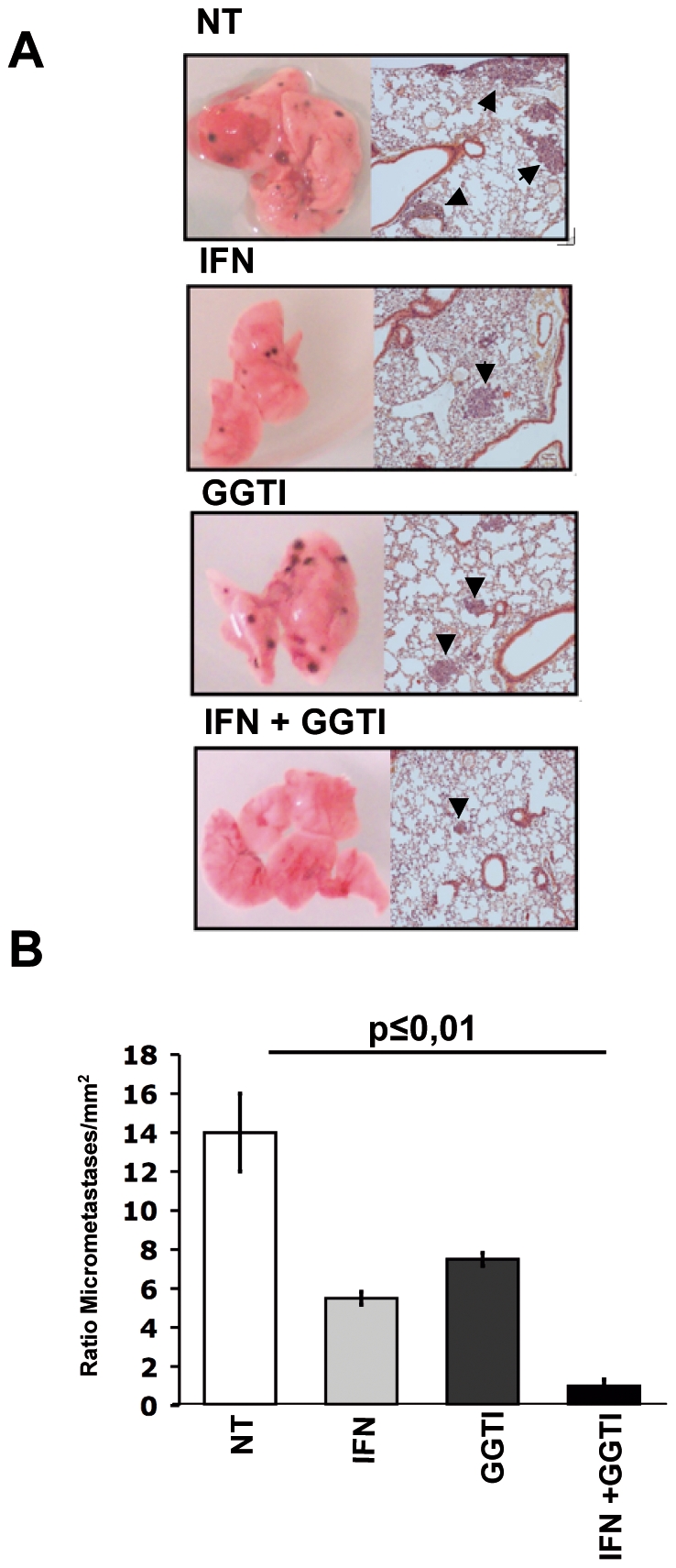
Syngeneic mice vaccination with mIFN-γ+GGTI-298 pretreated B16F10 cells induces resistance to pulmonary metastases implantation. Four groups of C57Bl/6 mice were vaccinated with untreated B16F10 cells (NT); mIFN-γ (50 IU/mL) (IFN); GGTI-298 (10 µM) (GGTI); or mIFN-γ (50 IU/mL) and GGTI-298 (10 µM) (IFN+GGTI). One month later, they were challenged intravenously with untreated B16F10 cells. (**A**) Metastases were screened macroscopically and microscopically as illustrated. (**B**) Lungs from the 4 groups of mice were slidded vertically and micro-metastases were counted and reported to the lung surfaces (mm^2^).

We next tested the ability of these pharmacologically pretreated B16F10 cells to induce, through vaccination, a protection against pulmonary metastases implantation. Again syngeneic mice were vaccinated as previously described and challenged intra-venously with 5×10^4^ B16F10 cells one month later. Four groups of mice were vaccinated with B16F10 cells either untreated, or pretreated with mIFN-γ (50 UI/mL), GGTI-298 (10 µM), or mIFN-γ (50 UI/mL) plus GGTI-298 (10 µM). All mice were sacrificed 15 days after tumor challenge and pulmonary macro- and micro-metastases were quantified as illustrated in Figure 2A and B. The results showed that vaccination with mIFN-γ plus GGTI-298 pretreated B16F10 cells is by far more efficient to protect against pulmonary metastases implantation (Figure 2B).

Altogether, these results show that pretreatment of B16F10 melanoma cells with a combination of mIFN-γ and GGTI-298 induces modifications of these cells such that they become immunogenic cells able to induce by vaccination protection against tumor growth and pulmonary metastases implantation.

### GGTI-298 Enhances hIFN-γ-Induced Expression of MHC-I but Not MHC-II in Human Melanoma Cells

**Figure 3:**
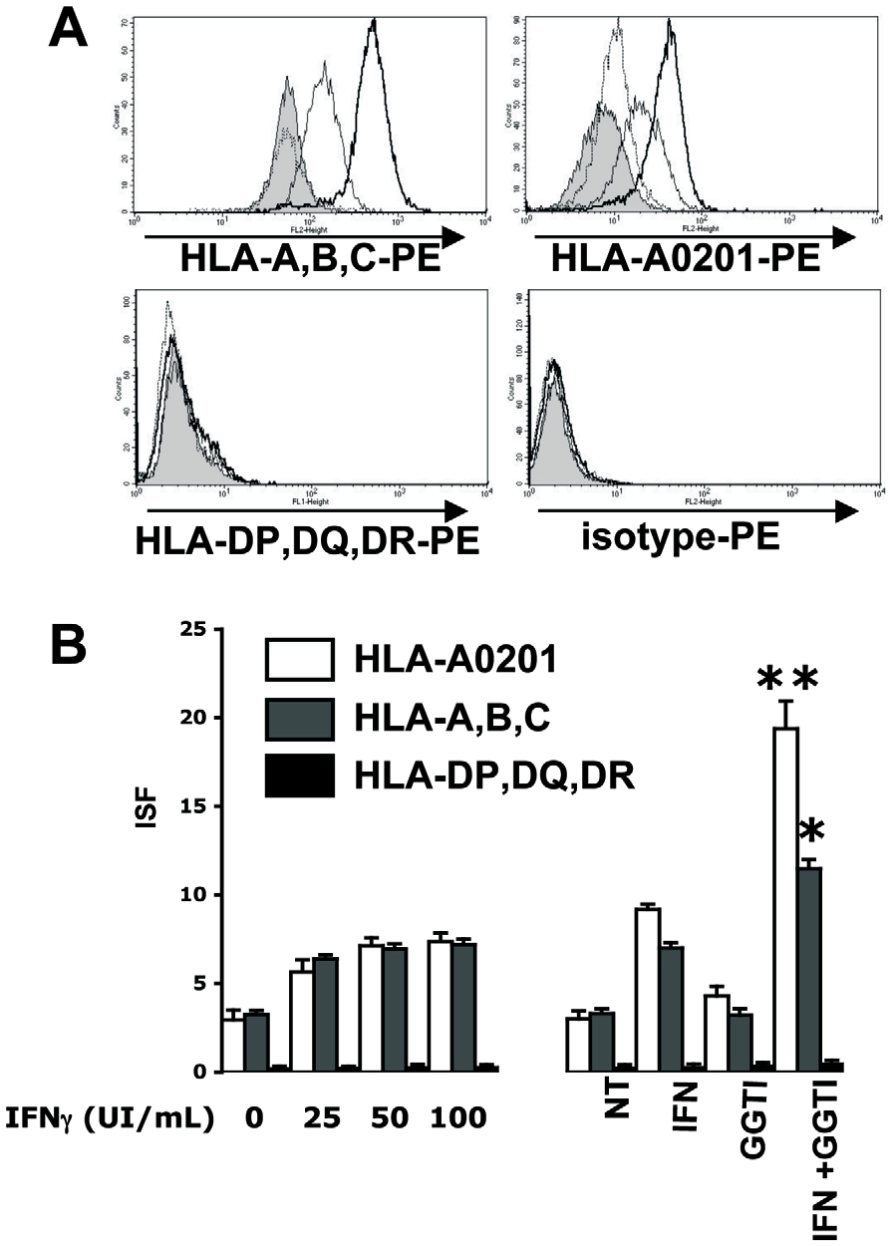
GGTI-298 enhances hIFN-γ-induced MHC-I expression on human melanoma cells. LB1319-MEL cells were grown for 4 days in culture medium (filled profiles) or in the presence of hIFN-γ alone (50 IU/ml) (thin lines) or GGTI-298 alone (10 µM) (dotted lines) or IFN-γ and GGTI-298 (thick lines). **A**) HLA-A,B,C ; HLA-A0201; HLA-DP,DQ,DR and isotypic controls expressions were measured by flow cytometry after staining with PE-conjugated mAbs. **B**) HLA-A0201 (White Column) HLA-A,B,C (Grey Column) and HLA-DP,DQ,DR (Black Column) membrane expressions were tested by cytofluorometry on LB1319-MEL cells after 4 days treatment with either increasing doses of hIFN-γ (0, 25, 50 and 100 IU/ml) or with the combination of hIFN-γ (50 IU/ml) and GGTI-298 (10 µM). Results are expressed in ISF, related to isotype controls, as indicated in Material and Methods. Data are representative of 3 independent experiments.

MHC molecules present peptides derived from TAA on tumor cell membranes. MHC-I presents peptides from cytoplasmic proteins that are recognized by cytotoxic T CD8+ lymphocytes, with responses leading to clinical tumor regression [3] [5] [6]. Thus, strong expression of MHC-I on tumor cell membranes favors tumor immune rejection. We tested the ability of *in vitro* treatment of human melanoma cells with hIFN-γ and GGTI-298 to enhance membrane MHC-I expression, and in particular the MHC-I allele able to present an immunodominant TAA peptide. For this purpose, we cultivated LB1319-MEL cells, which are spontaneously HLA-ABC positive and express HLA-A0201 molecules, for 4 days with GGTI-298 (10 µM), either alone or with hIFN-γ (50 IU/mL). A concentration of 10 µM of GGTI-298 prevented protein isoprenylation and was only weakly toxic in our culture conditions (data not shown). Higher concentrations were not used due to their cellular toxicity. As shown in Figure 3, despite a strong constitutive expression of MHC-I and HLA-A0201, GGTI-298 treatment enhanced significantly the hIFN-γ-induced membrane expression of these molecules in LB1319-MEL cells (p<0,001). In this cell line expression of HLA-ABC and HLA-A0201 increased in a dose-dependent manner in hIFN-γ-treated cells, reaching a steady-state level at 50 IU/mL, but these expressions were further significantly enhanced by addition of GGTI-298 (10 µM). However, GGTI-298 alone (10 µM) did not significantly modify expression of MHC-I and in particular HLA-A0201 (Figures 3A and 3B).

MHC-II molecules present peptides derived primarily from extra-cellular proteins, and these are recognized by auxiliary CD4+ T lymphocytes. Some of these CD4+ T cells “help” CD8+ T lymphocytes via cytokine production. With few exceptions, tumor cells do not express MHC-II molecules. We tested the ability of *in vitro* treatment with hIFN-γ alone or with GGTI-298, to induce membrane MHC-II (HLA-DP,-DQ,-DR) expression in MHC-II-negative LB1319-MEL cells. As shown in Figure 3A and 3B, treatment with hIFN-γ (50 IU/mL) for 4 days did not induce HLA-DP,-DQ,-DR expression; nor did higher concentrations up to 100 IU/mL or 500 IU/mL (data not shown). Moreover addition of GGTI-298 (10 µM) did not modify MHC-II expression.

Overall, these results show that GGTase I inhibition further enhanced hIFN-γ–induced MHC-I membrane expression in these MHC-I positive human melanoma cell lines. In contrast, these cells remained MHC-II-negative after such treatments.

### GGTI-298 Enhances hIFN-γ-Induced TAP1 and TAP2 in LB1319-MEL Cells

**Figure 4:**
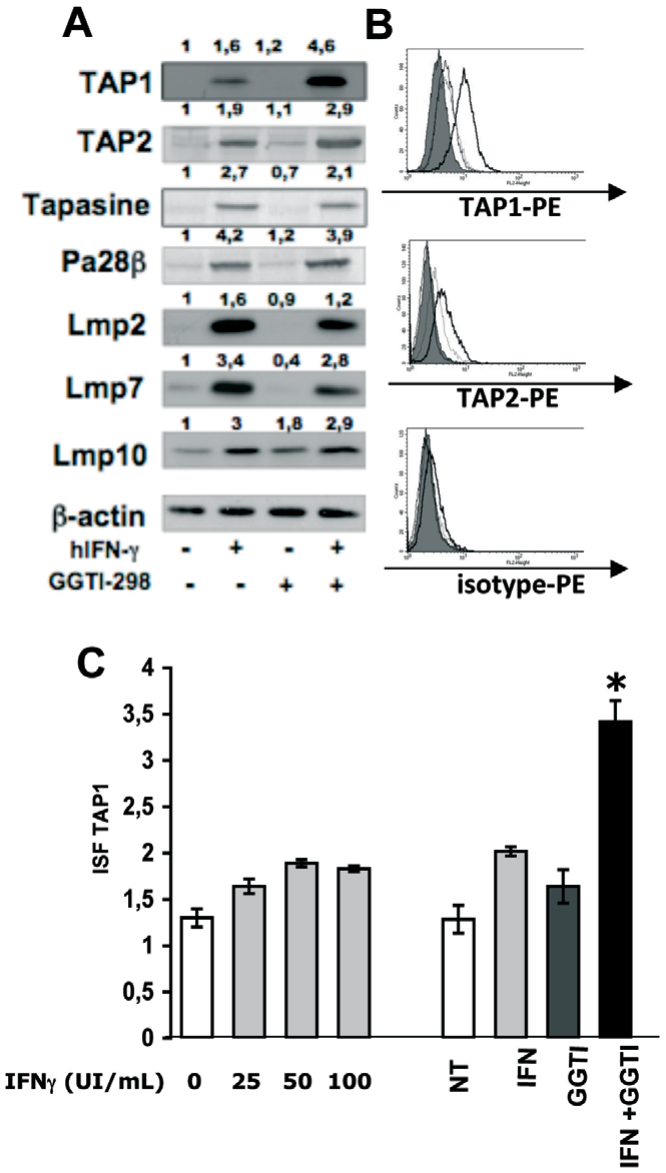
On LB1319-MEL cells, GGTI-298 enhances hIFN-γ-induced TAP1 and TAP2 expressions. LB1319-MEL cells were cultivated for 4 days in the presence or in the absence of hIFN-γ (50 IU/ml) and/or GGTI-298 (10 µM) as indicated. **A**) The expression of molecules implicated in the MHC-I Ag processing pathway were tested in these treated tumor cells. β-actin was used as protein loaded control. The expressions were quantified by calculating the ratio between the protein of interest and the β-actin. We defined the ratio of relevant protein over β-actin for untreated cells equal to 1. **B**) Expressions of TAP1 and TAP2 proteins in these *in vitro* treated and permeabilized LB1319-MEL were tested by flow cytometry using TAP1 and TAP2 specific mAbs and a PE-conjugated secondary Ab. **C**) TAP1 membrane expression was also tested by cytofluorometry on LB1319-MEL cells after 4 days treatment with either increasing doses of hIFN-γ (0, 25, 50 and 100 IU/ml) or with the combination of hIFN-γ (50 IU/ml) and GGTI-298 (10 µM). Results are expressed in ISF, related to isotype controls, as indicated in Material and Methods. Data illustrated are representative of 3 independent experiments.

Peptide presentation by MHC-I antigens is linked to the coordinated activity of multiple components of the MHC-I antigen processing pathway (APP), including the peptide transporters TAP1 and TAP2 [2], the chaperon protein tapasin, and the subunits of the multicatalytic proteasome (PA28, LMP2, LMP7, and LMP10). Seliger et al. [20] demonstrated that the defect in MHC-I expression in the B16F10 murine tumor model was linked to downregulation of proteins in the MHC-I APP, which could be corrected by mIFN-γ administration. We previously demonstrated in the same murine model that treatment with GGTI-298 enhances mIFN-γ-induced TAP1 expression [1]. In the current study we used the LB1319-MEL human melanoma model to investigate the effects of 4 days of *in vitro* treatment with hIFN-γ (50 IU/mL) and/or GGTI-298 (10 µM) on the expression of TAP1, TAP2, tapasin, PA28, LMP2, LMP7, and LMP10 proteins (Figure 4).

As observed in the murine model, treatment with hIFN-γ alone up-regulated the expression of all measured proteins of the MHC-I APP, while treatment with GGTI-298 alone had no significant effect (Figure 4A). When LB1319-MEL cells were jointly treated with GGTI-298 and hIFN-γ, TAP1 and TAP2 expression underwent a 3-fold and 1,5 fold increase, respectively, relative to expression following treatment with hIFN-γ alone (Figure 4A). Addition of GGTI-298 had no significant effect on the hIFN-γ-induced expression of the other tested proteins of the MHC-I APP (Figure 4A).

The results obtained by western blotting of TAP1 and TAP2 were confirmed by flow cytometry in LB1319-MEL cells treated with hIFN-γ and GGTI-298, permeabilized, and stained with anti-TAP1 or anti-TAP-2 antibodies (Figure 4B). As shown in Fig.4C, TAP1 expression increased dose-dependently in hIFN-γ-treated cells, reaching a steady-state level at 50 IU/mL; addition of GGTI-298 (10 µM) further increased expression. This increase is statistically significant with a p<0,05.

Collectively, these results show that GGTase I inhibition enhances hIFN-γ-induced TAP1 and TAP2 expression in this human melanoma cell line.

### In the LB1319-MEL Cells, GGTI-298 Enhances the Expression of a Stimulatory Molecule CD86 and Down Regulates the IFN-γ-Induced Expression of an Inhibitory Molecule PD-1L

**Figure 5:**
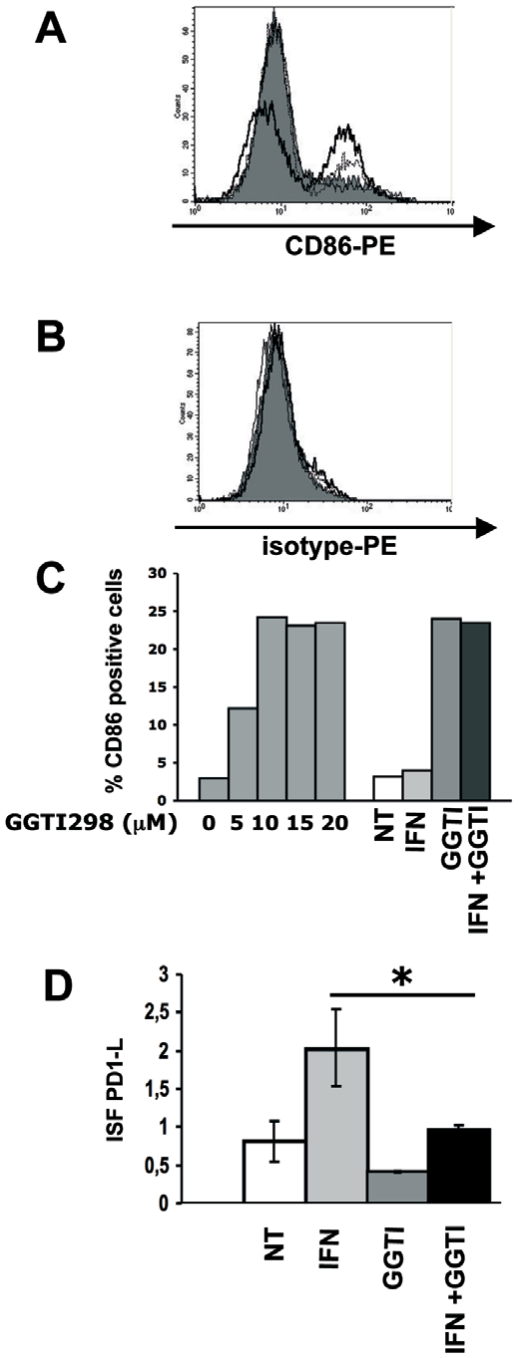
On LB1319-MEL cells, GGTI-298 induces enhancement of CD86 and reduction of IFN-γ-induced PD-1L expression. Membrane expression of CD86 (**A**) and its isotype control (**B**) was determined by flow cytometry with PE-conjugated specific antibodies on LB1319-MEL cells after 4 days *in vitro* treatment with medium alone (filled profiles); hIFN-γ (50 IU/mL) alone (thin lines); GGTI-298 (10 µM) alone (dotted lines); or the combination of both (thick lines). Data are representative of three independent experiments. **C**) CD86 membrane expression was also tested by cytofluorometry on LB1319-MEL cells after 4 days treatment with either increasing doses of GGTI-298 (0, 5,10, 15 and 20 µM) or with the combination of hIFN-γ (50 IU/ml) and GGTI-298 (10 µM). Results are expressed in percentage of CD86 positive cells. D) Membrane expression of inhibitory molecule PD-1L was determined by flow cytometry with PE-conjugated specific Ab on LB1319-MEL cells after 4 days *in vitro* treatment with or without hIFN-γ (50 IU/ml) and/or GGTI-298 (10 µM). Results are expressed in ISF, related to isotype controls, as indicated in Material and Methods. Data are representative of 3 independent experiments.

There are two families of costimulatory molecules expressed in antigen presenting cells (APCs) and some tumor cells: B7 and TNF/TNF receptor (TNF-R). Previously [1] we reported that *in vitro* treatment with GGTI-298 induces a dose-dependent increase in the membrane expression of CD80 and CD86 costimulatory molecules in the murine melanoma cells B16F10 and an increase in CD86 in two human melanoma cell lines, including LB1319-MEL. Here we confirmed that LB1319-MEL cells spontaneously express the costimulatory molecule CD86, and that this expression is unaffected by hIFN-γ treatment alone but strongly enhanced following treatment with GGTI-298 alone or in combination with hIFN-γ (Figure 5A). CD86 expression increased dose-dependently in GGTI-298-treated cells, reaching a steady-state level at 10 µM. Addition of hIFN-γ (50 IU/mL) did not induce further increased expression (Figure 5C).

Furthermore, FACS analysis showed that LB1319-MEL cells spontaneously express in their membrane the inhibitory molecule PD-1L [21]. Its expression was up regulated by hIFN-γ treatment and significantly (p<0,05) down regulated by GGTI-298 addition (Figure 5D).

Altogether, these results show that GGTI-298 treatment modifies the level of expression of costimulatory molecules which are spontaneously expressed on the membrane of LB1319-MEL melanoma cells. Interestingly, it appears that this pharmacological treatment increases the immunogenicity of the tumor cells because it enhances the expression of the stimulatory CD86 molecules and it down regulates the IFN-γ-induced expression of the inhibitory molecule PD-1L.

### *In Vitro* Stimulation of PBMC from Healthy Donors Using LB1319-MEL Cells Pretreated with hIFN-γ and GGTI-298 Induces CD8 T Lymphocyte Activation

**Figure 6:**
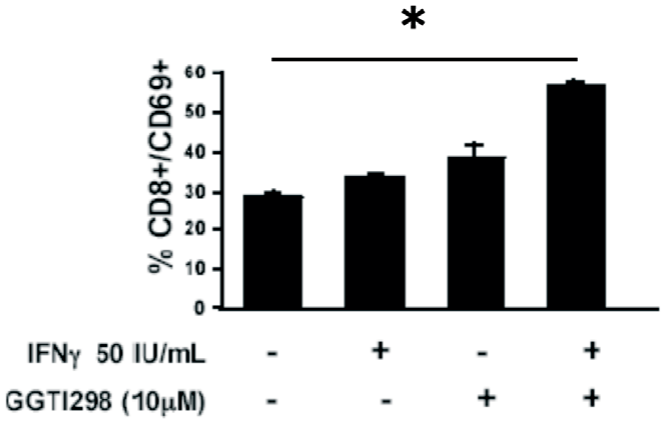
Enhanced activation of CD8+ T lymphocytes in PBMC co-cultivated with LB1319-MEL cells pre-treated with hIFN-γ+GGTI-298. PBMC from HD HLA-A0201 were stimulated twice with LB1319-MEL cells either untreated; or pretreated with hIFN-γ (50 IU/mL); GGTI-298 (10 µM); or hIFN-γ (50 IU/mL) plus GGTI-298 (10 µM). CD8+ activation was evaluated by CD69 membrane expression. Experiments were performed in triplicate with PBMC from one healthy donor representative of one other giving comparable results.

We next wished to test the ability of this combined treatment to convert LB1319-MEL melanoma cells into stimulator cells able to induce an enhanced *in vitro* activation of lymphocytes and particularly of CD8 T lymphocytes. For this, we twice stimulated PBMC from 3 healthy HLA-A0201 donors (HD) *in vitro* with LB1319-MEL melanoma cells which had been pretreated with hIFN-γ and/or GGTI-298, washed twice and then irradiated as described in Materials and Methods. Activation of different lymphocytes subpopulations was analyzed by flow cytometry. Stimulation with hIFN-γ and especially hIFN-γ associated with GGTI-298 pretreated tumor cells enhanced the activation of CD8+ lymphocytes. These activated cells expressed the activation marker CD69, which is expressed rapidly upon activation [22]. In these conditions CD4+ and NK cells activation remains unchanged (data not shown). But we detected a 2-fold increase in CD8+/CD69+ cells following stimulation of the PBMC with LB1319-MEL cells pretreated with hIFN-γ and GGTI-298 versus hIFN-γ alone (Figure 6). This shows that pretreatment of LB1319-MEL cells with hIFN-γ and GGTI-298 increases their efficiency to activate CD8 T lymphocytes from PBMC of HLA-A0201 HD *in vitro*.

### *In Vitro* Stimulation of PBMC from HD Using hIFN-γ and GGTI-298-Treated LB1319-MEL Melanoma Cells Induces IFN-γ Release

In an effort to determine whether LB1319-MEL cells pretreated with hIFN-γ and GGTI-298 can become stimulatory cells capable of inducing the functional activation of co-cultivated lymphocytes, we analyzed the cytokine production in the co-cultures. By multiplex analysis for IL-2, IL-4, IL-10, and IFN-γ (Linco Multiplex), we investigated the TH1/TH2 profile of cytokines released by the stimulated cells. IL-2 and IFN-γ secretion is representative of the TH1 profile, mainly implicated in cellular immune responses, whereas IL-4 and IL-10 secretion is associated with the TH2 profile, mainly implicated in the humoral immune response and/or negative regulation of the immune response [23]. Secretion levels were examined in the supernatants of co-cultures 24 h after the second stimulation of the PBMC with the pretreated tumor cells.

A significant increase in hIFN-γ secretion was observed when the LB1319-MEL melanoma stimulatory cells were pretreated with hIFN-γ alone, which was further enhanced following pretreatment with a combination of hIFN-γ and GGTI-298 (Table 1). These stimulations also induced an increased secretion of hIL-2, the concentration of which only reached moderate levels in the supernatants, perhaps due to autocrine consumption by proliferating T lymphocytes (Table 1). These results suggest a TH1 cytokine secretion in our culture conditions.

**Table 1:** Enhanced IFN-γ secretion by PBMC co-cultivated with LB1319-MEL cells pre-treated *in vitro* with hIFN-γ+GGTI-298.

	Cytokines concentration (+/- SD)			
	IL-2	IFN-γ	IL-4	IL-10
**NT**	1,5 (+−/1)	**71,1 (+/−5)**	5,98 (+/−3)	37,6 (+/−6)
**IFN**	3,9 (+/−2)	**206 (+/−24)**	42,7 (+/−3)	38,8 (+/−2)
**GGTI**	1,7 (+/−1)	**148 (+/−19)**	18,3 (+/−6)	39,3 (+/−7)
**IFN+GGTI**	4,2 (+/−1)	**418 (+/−4)**	36,3 (+/−1)	37,7 (+/−7)

PBMC from 3 HLA-A0201 HD were stimulated twice *in vitro* with LB1319-MEL cells untreated (NT); or pretreated with 50 UI/mL hIFN-γ (IFN); 10 µM GGTI-298 (GGTI); or the combination of 50 UI/mL hIFN-γ and 10 µM GGTI-298 (IFN+GGTI) as indicated. 24h after the second *in vitro* stimulation, cytokine secretions in the culture supernatants were measured by Multi-Plex human cytokine assays. Results are illustrated in pg/mL for secretions of IFN-γ, IL-2, IL-4 and IL-10. Data are shown as mean (+/− standard deviation) of duplicate values and were obtained from one donor representative of the two others giving comparable results.

However, pretreatment of the LB1319-MEL stimulatory cells with hIFN-γ alone also induced an increase in hIL-4 secretion by the co-cultivated cells, whereas the combination with GGTI-298 showed no enhancement (Table 1). Moreover, no significant changes in IL-10 levels were observed in the different co-culture conditions (Table 1).

Altogether, these results do not reveal a strict TH1 or TH2 profile of cytokine secretion, but they do show that the pretreatment of LB1319-MEL cells with hIFN-γ and GGTI-298 induces modifications of these cells such that they induce increased hIFN-γ secretion by the cells present in the co-culture.

### LB1319-MEL Cells Pre-Treated *In Vitro* with hIFN-γ and GGTI-298 Enhance Specific Anti-Melanoma Cytotoxic Activity of Co-Cultivated PBMC

**Figure 7:**
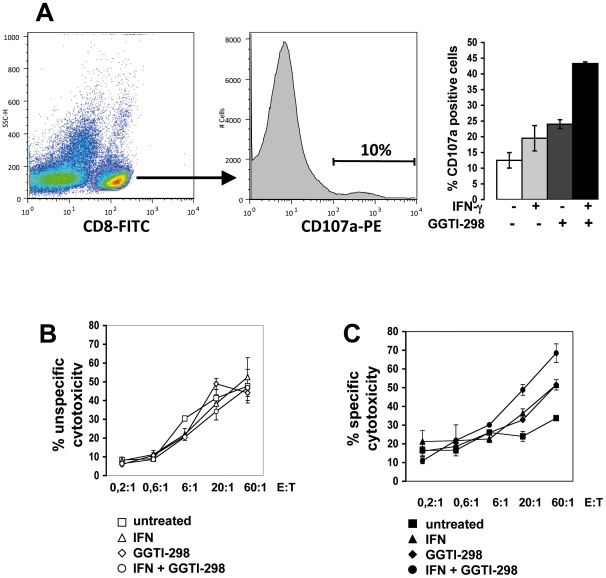
Enhanced specific cytotoxic activity in PBMC co-cultivated with LB1319-MEL cells pre-treated with hIFN-γ+GGTI-298. Cytotoxic activities of *in vitro* stimulated PBMC were tested after two stimulations with LB1319-MEL cells either untreated or pretreated with hIFN-γ (50 IU/mL); GGTI-298 (10 µM); or hIFN-γ (50 IU/mL) plus GGTI-298 (10 µM). **A**) Cytotoxicity was evaluated by CD107a expression on CD8+ gated T lymphocytes. Cytotoxic activity was also tested on an unspecific target cell: **B**) Jurkat and the specific one: **C**) LB1319-MEL. Experiments were performed in triplicate with PBMC from one healthy donor representative of one other giving comparable results.

IFN-γ can be secreted by NK cells or CD8 T effector lymphocytes and is linked to efficient cytotoxic activity. We next wished to determine whether pretreated LB1319-MEL cells become stimulatory cells capable of inducing anti-melanoma cytotoxic effector cells from the co-cultivated PBMC of HLA-A0201 compatible HD. The cytotoxic activity of these stimulated PBMC was then tested. As cytotoxic cells express the lysosomal membrane protein (CD107a) [24], the expression of this molecule was analyzed among the CD8 positive cells present in these stimulated PBMC. As shown in Figure 7A, CD107a positive cells are significantly increased in the co-culture stimulated by LB1319-MEL cells pretreated by IFN-γ and GGTI-298. Moreover the cytotoxic activity of these stimulated PBMC was also tested directly by their killing capacity on both an unspecific target cell line Jurkat and on the specific one LB1319-MEL. The effector cells obtained after the co-culture had similar non-specific cytotoxic capacity whatever the pretreatment of the stimulator LB1319-MEL melanoma cells (Figure 7B). On the contrary, the 4 different pretreatments induced different levels of specific cytotoxic activity detected on untreated LB1319-MEL target cells (Figure 7C). Indeed we found a significantly increased cytotoxicity following the stimulation of PBMC by the combined hIFN-γ plus GGTI-298 pretreated melanoma cells. The pretreatment with hIFN-γ or GGTI-298 alone induced intermediate lytic activity and untreated melanoma cells induced very low specific cytotoxicity (Figure 7C).

These results show that pretreatment of LB1319-MEL melanoma cells with a combination of hIFN-γ and GGTI-298 induces modifications of these cells such that they become stimulator cells able to enhance specific anti-melanoma cytotoxic effectors in co-cultivated HLA-compatible PBMC from HD.

### *In Vitro* Stimulation of PBMC from HD Using hIFN-γ and GGTI-298-Pretreated LB1319-MEL Cells Increases the Proliferation of Tumor-Specific CD8 T Lymphocytes Labeled with MART-1/HLA-A2 Tetramers

**Figure 8:**
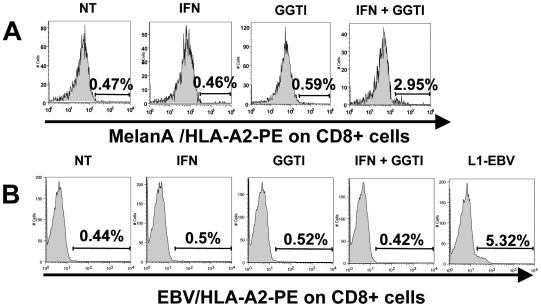
PBMC stimulation with hIFN-γ+GGTI-298 pretreated LB1319-MEL cells induces specific cells proliferation, labeled with MART-1/HLA-A2 tetramers. **A**) HLA-A0201 positive PBMC from one representative HD were stimulated twice *in vitro* with LB1319-MEL cells either untreated (NT) or pretreated with 50 UI/mL hIFN-γ (IFN), or 10 µM GGTI-298 (GGTI), or with both hIFN-γ and GGTI-298 (IFN+GGTI). At the end of the culture period, specific TCR MelanA / HLA-A2 expression was evaluated on CD8+ gated T lymphocytes. **B**) HLA-A0201 positive PBMC from the same HD were stimulated twice *in vitro* with LB1319-MEL cells either untreated (NT) or pretreated with 50 UI/mL hIFN-γ (IFN), or 10 µM GGTI-298 (GGTI), or with both hIFN-γ and GGTI-298 (IFN+GGTI) or with irradiated (HLA-A0201) L1-EBV cells. At the end of the culture period, specific TCR EBV/HLA-A2 expression was evaluated on CD8+ gated T lymphocytes.

The specific cytotoxicity observed after co-cultivating PBMC with LB1319-MEL cells pretreated with the combination of hIFN-γ and GGTI-298, suggests that these melanoma cells develop the ability to induce a specific anti-tumor immune response *in vitro*, likely linked to the increased proliferation of specific CD8 T lymphocytes. To test this hypothesis, PBMC from 3 HLA-A0201 HD were twice stimulated *in vitro* with LB1319-MEL cells pretreated with hIFN-γ and/or GGTI-298 as before. The T lymphocyte subpopulations present in the co-culture were then analyzed by flow cytometry. The LB1319-MEL melanoma cell line expresses peptides on the membrane, obtained from MelanA/MART-1 proteins and associated with HLA-A0201 molecules, in particular the immunodominant peptide EAAGIGILTV [19]. LB1319-MEL specific CD8 T lymphocytes should therefore express TCR specific for this HLA-A0201-presented peptide, which can be labeled with MART-1/HLA-A0201 tetramers. We observed a significant increase of the CD8 subpopulation labeled with these tetramers in the 3 tested donors (Table 2 and illustrated for one HD in Figure 8A). On the contrary, these stimulated PBMC have similar levels of non-specific TCR, detected with EBV/HLA0201 tetramers, whatever the pretreatment of the stimulator LB1319-MEL cells as illustrated in Figure 8B. However, the control PBMC cultures stimulated by an HLA-A0201 EBV immortalized lymphoblastoid cell line (L1-EBV) were strongly positive with these EBV/HLA0201 tetramers (Figure 8B).

**Table 2:** PBMC stimulation with hIFN-γ+GGTI-298 pretreated LB1319-MEL cells induces CD8+ and MART-1/HLA-A0201+ specific cells proliferation.

	Percentage (%) of cells CD8+ and MART1/HLA-A2+			Fold Induction (FI) of cells CD8+ and MART1/HLA-A2+		
	HD1	HD2	HD3	HD1	HD2	HD3
**NT**	1,5	0,29	0,07	1	1	1
**IFN**	2,7	0,39	0,11	1,8	1,34	1,57
**GGTI**	1,03	0,65	0,03	0,68	2,24	0,42
**IFN+GGTI**	4,8	1,13	0,35	3,2	3,9	5

PBMC from 3 HLA-A0201 HD were stimulated twice *in vitro* with LB1319-MEL cells untreated (NT); or pretreated with 50 UI/mL hIFN-γ (IFN); 10 µM GGTI-298 (GGTI); or with a combination of 50 UI/mL hIFN-γ and 10 µM GGTI-298 (IFN+GGTI) as indicated. At the end of the culture period, PBMC were stained with FITC-conjugated anti-CD8 monoclonal antibody and with PE-conjugated MART-1/HLA-A2 tetramers. Percentages of double positive CD8+ and MART-1/HLA-A2 tetramers+ PBMC are shown. Fold induction between double positive CD8+ and MART-1/HLA-A2+ cells obtained after co-culture with untreated or pre-treated LB1319-MEL cells is shown. We defined the percentage of double positive cells obtained with the untreated melanoma cells (NT) as equal to 1 and compared this to the values obtained in the other culture conditions.

These results show that *in vitro* stimulation of PBMC from HLA-A0201 HD, with hIFN-γ and GGTI-298 pretreated LB1319-MEL cells, induces the enhancement of the specific anti-tumor CD8 T lymphocyte subpopulation.

## Discussion

Melanoma is a highly lethal cutaneous tumor, killing affected patients through multiple, poorly immunogenic metastases. Several previous studies have investigated vaccine-based and curative approaches for inducing an immune response against melanoma cells. Different protocols have been investigated for obtaining cellular tools necessary for vaccination or curative adoptive transfer, with the intent of stimulating PBMC or tumor infiltrating lymphocytes (TIL) from the patients *in vitro* before re-effusion [3] [19]. The use of pulsed DC [6] or tumor-DC hybrids [7] is very attractive however the development of highly immunogenic melanoma cell lines also represents another interesting approach. We recently reported that a combination of GGTI-298 and mIFN-γ increased the immunogenicity of a murine melanoma cell line[1]. Using the melanoma cell line B16F10, we showed that treatment with GGTI-298 and mIFN-γ inhibited tumor growth *in vivo* by a mechanism involving the activation of specific CD8 T lymphocytes.

Furthermore, in the present study, we showed that vaccination with IFN-γ and GGTI-298 pretreated B16F10 melanoma cells reduces tumor recurrence and also exert an anti-metastatic effect. Indeed, *in vivo* vaccination of syngeneic mice with pharmacologically pretreated melanoma cells induces tumor growth slowing down and reduction of pulmonary metastases implantation.

Moreover, we have demonstrated here that the same treatment applied to a cutaneous metastatic human melanoma cell lines, modifies the expression of molecules in the cellular membrane in a manner favoring a specific anti-tumor immune response. Our results indicate that GGTI-298 treatment inhibits the activity of a geranylgeranylated protein involved in hIFN-γ induced HLA-A0201 expression. As previously observed in the murine melanoma model, the GGTase I inhibition enhances IFN-γ-induced MHC class I expression through a mechanism involving TAP1 and TAP2 over-expression.

The human melanoma cell line used is obtained from a cutaneous metastasis and it express spontaneously the costimulatory molecule CD86 on its membrane, which is in agreement with findings of Hersey *et al.* showing that few melanoma metastases cells express CD80 or CD86 [25]. So, our results show that GGTI-298 treatment does not induce but increases CD86 expression on melanoma cell membranes. This suggests that GGTI-298 may increase the membrane localization rather than expression levels of CD86, as we recently demonstrated with FasL protein on the murine melanoma cell line B16F10 [26].

Due to its expression of an HLA-I haplotype (HLA-A0201) and a TAA (MelanA/MART-1), both expressed on a large proportion of human melanoma cells [27], the LB1319-MEL melanoma cell line is an interesting and representative cell line. DC are the most potent and efficient APC in the immune system. They express high levels of MHC-I- and MHC-II-encoded gene products and costimulatory molecules on their membrane. By triggering the over-expression of MHC-I molecules and costimulatory molecules, *in vitro* treatment with a combination of hIFN-γ and GGTI-298 modifies LB1319-MEL melanoma cells such that they acquire some characteristics of stimulatory APC and become more efficient stimulator cells able to activate CD8 T lymphocytes from PBMC of HLA-compatible HD.

Our results show that the modifications induced by the pretreatment of LB1319-MEL melanoma cells with hIFN-γ and GGTI-298 promote an anti-tumor immune response by increasing the number of specific CD8 T lymphocytes labeled with MART-1/HLA-A2 tetramers. Furthermore, *in vitro* stimulated PBMC are able to secrete significant amounts of hIFN-γ and develop specific anti-melanoma cytotoxic activity. These abilities render them suitable for anti-tumor immune surveillance [18] [27].

In conclusion, according to the results obtained with the murine and the human melanoma models, a combined *ex-vivo* pharmacological treatment with hIFN-γ and GGTI-298, may represent a promising approach to produce specific anti-melanoma effector cells and immunogenic melanoma cells suitable for vaccination.
